# Left atrial Thrombus formation after discontinuation of anticoagulation in patient with severe bioprosthetic mitral stenosis

**DOI:** 10.1186/s12872-023-03644-7

**Published:** 2023-12-14

**Authors:** Ryan C. King, Tobin Mathew, Stella Whang, Ned Premyodhin, Pranav Patel

**Affiliations:** Department of Medicine, Division of Cardiology, University of California, Irvine Medical Center, 101 The City Drive, S, Orange, CA 92868 USA

**Keywords:** Mitral valve stenosis, Bioprosthetic valve, Left atrial thrombus, Anticoagulation, Warfarin, Hypercoagulability

## Abstract

**Background:**

Mitral valve stenosis can be a highly symptomatic condition with significant complications if left untreated. In such cases, mitral valve replacement with a bioprosthetic or mechanical valve may be a viable solution to prevent progressive disease. Current guidelines do not recommend continued anticoagulation beyond 6 months for patients who have undergone bioprosthetic valve replacement without a separate indication for anticoagulation. With this case discussion we aim to 1) Review the current indications for anticoagulation for bioprosthetic mitral valves in patients without atrial fibrillation and 2) Discuss the constellation of comorbidities that may affect the decision to begin anticoagulation therapy.

**Case presentation:**

We present a case describing a 55-year-old male with end-stage renal disease, coronary artery disease with coronary artery bypass graft surgery, and bioprosthetic mitral valve replacement 2 years prior with rapid degeneration of the replaced valve and on warfarin without a clear indication for anticoagulation. The patient was admitted for symptomatic, severe mitral stenosis and consideration of transcatheter mitral valve-in-valve replacement. During hospital admission, warfarin was discontinued and replaced with prophylactic anticoagulation. However, 8 days after warfarin cessation an intraoperative transesophageal echocardiography revealed a newly developed large left atrial thrombus leading to cancellation of the planned operation.

**Conclusions:**

This patient developed a left atrial thrombus after discontinuing warfarin in the setting of rapidly deteriorating bioprosthetic valve stenosis and vascular comorbidities. The decision to discontinue warfarin was made in concordance with current guidelines, which do not indicate systemic anticoagulation post 3–6 months after bioprosthetic valve replacement without separate indication for anticoagulation. This case identifies the need to investigate rebound hypercoagulability and further risk stratify comorbidities which may independently increase the risk of clot formation in the setting of severe mitral valve stenosis.

## Background

According to current valvular heart disease guidelines, mitral stenosis is not an indication for therapeutic anticoagulation in the absence of atrial fibrillation or additional indications. Moreover, without further indications for anticoagulation a short course of aspirin with warfarin is typically recommended for three to 6 months following bioprosthetic mitral replacement [1]. Further investigation and risk stratification regarding therapeutic anticoagulation in the setting of isolated mitral stenosis is warranted in patients living with risk factors associated with hypercoagulability.

## Case presentation

**Fig. 1:**
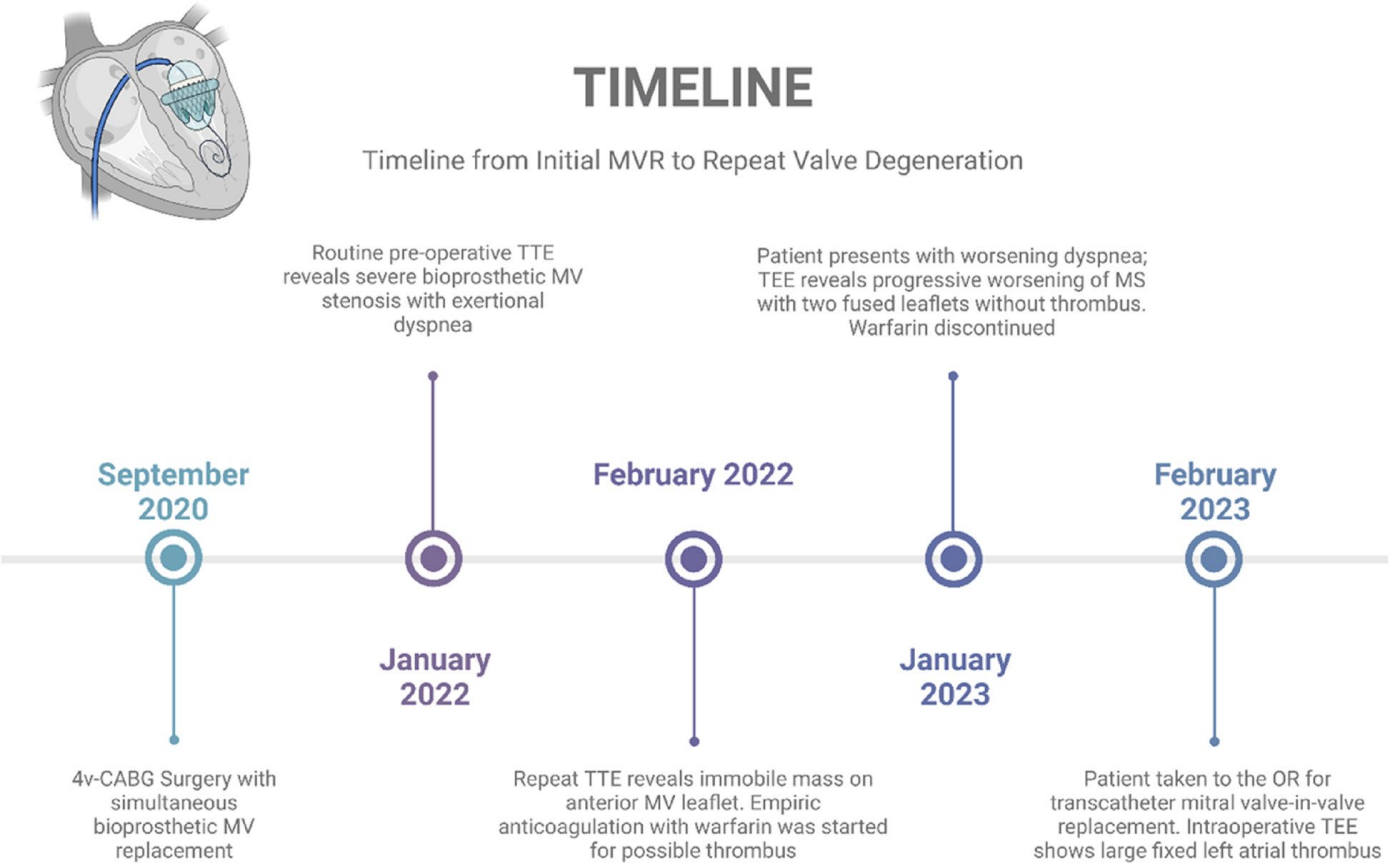
Timeline from initial mitral valve replacement to bioprosthetic valve degeneration and thrombus discovery. Abbreviations: Mitral valve (MV); MV replacement (MVR); Transthoracic echocardiogram (TTE); Four-vessel coronary artery bypass graft (4v-CABG); Mitral Stenosis (MS); Transesophageal echocardiogram (TEE); operating room (OR). Figure created with Biorender.com

A 55-year-old male with ESRD on hemodialysis secondary to longstanding hypertension, and CAD underwent simultaneous 4v-CABG, surgical bioprosthetic MV replacement (Edwards 27 mm), and left atrial appendage ligation repair in Sept 2020 (Fig. 1). Post-operative transesophageal echocardiography (TEE) noted a mean MV gradient of 3 mmHg at a normal heart rate under general anesthesia with an ejection fraction (EF) of 40–45%. There was also trace mitral regurgitation within expected limits of the prosthesis without evidence of perivalvular leak. In addition, there was no sign of aortic stenosis or aortic regurgitation with normal aortic pressure and velocity noted postoperatively. The patient then completed a short-term course of warfarin and aspirin for 3 months. He presented 2 years later in January 2022 after a routine preoperative transthoracic echocardiography (TTE) for an elective total hip arthroplasty revealed severe stenosis of the bioprosthetic mitral valve with mean gradient of 11 mmHg, heart rate (HR) 70 beats per minute (bpm), EF 55%, MV pressure half time 115 ms, and effective orifice mitral valve area 1.9 cm^2^ (Table 1). He experienced progressive exertional dyspnea and chest tightness with limited ambulation of 10 ft.

**Table 1:** Echocardiogram parameters from time of CABG to discovery of left atrial thrombus

	Post op 9/2020 (TEE)	January 2022 (TTE)	January 2023 (TEE)	February 2023 (TEE)
Heart Rate (bpm)	Normal under general anesthesia	70	80	80
Mean Mitral Gradient (mmHg)	3	11.0	21.0	19.0
Mitral valve area (cm^2^)	Not available	1.91	0.48	Not available
Mitral valve diastolic-pressure half-time (msec)	Not available	115.25	454.29	Not available
LVEF (%)	40–45%	55	Moderately decreased	Not available

Available echocardiogram data from the initial post-operative transesophageal echocardiogram (TEE) to discovery of left atrial thrombus

**Fig. 2:**
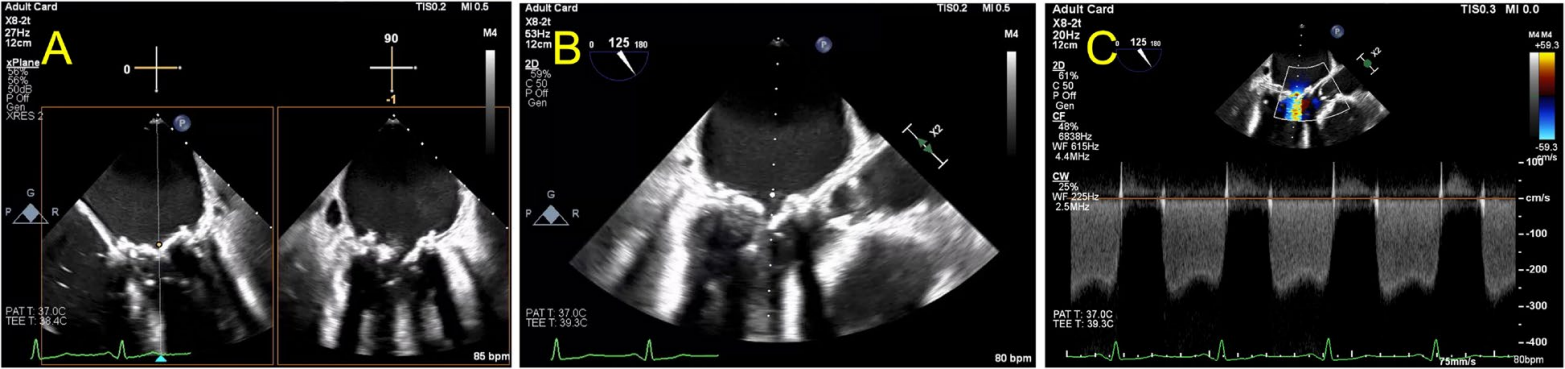
Pre-thrombus echocardiographic images of mid-esophageal 4 chamber and long axis view of bioprosthetic mitral valve. Pre-thrombus transesophageal echocardiogram (TEE) images of the bioprosthetic mitral valve in end-diastole demonstrating restricted leaflet motion in the **A** mid-esophageal 4 chamber view at 0 degrees and 90 degrees (biplane), **B** long axis view at 125 degrees and **C** long axis view with continuous wave Doppler envelope consistent with severe mitral stenosis with peak velocity of 2.5 m/sec

**Fig. 3:**
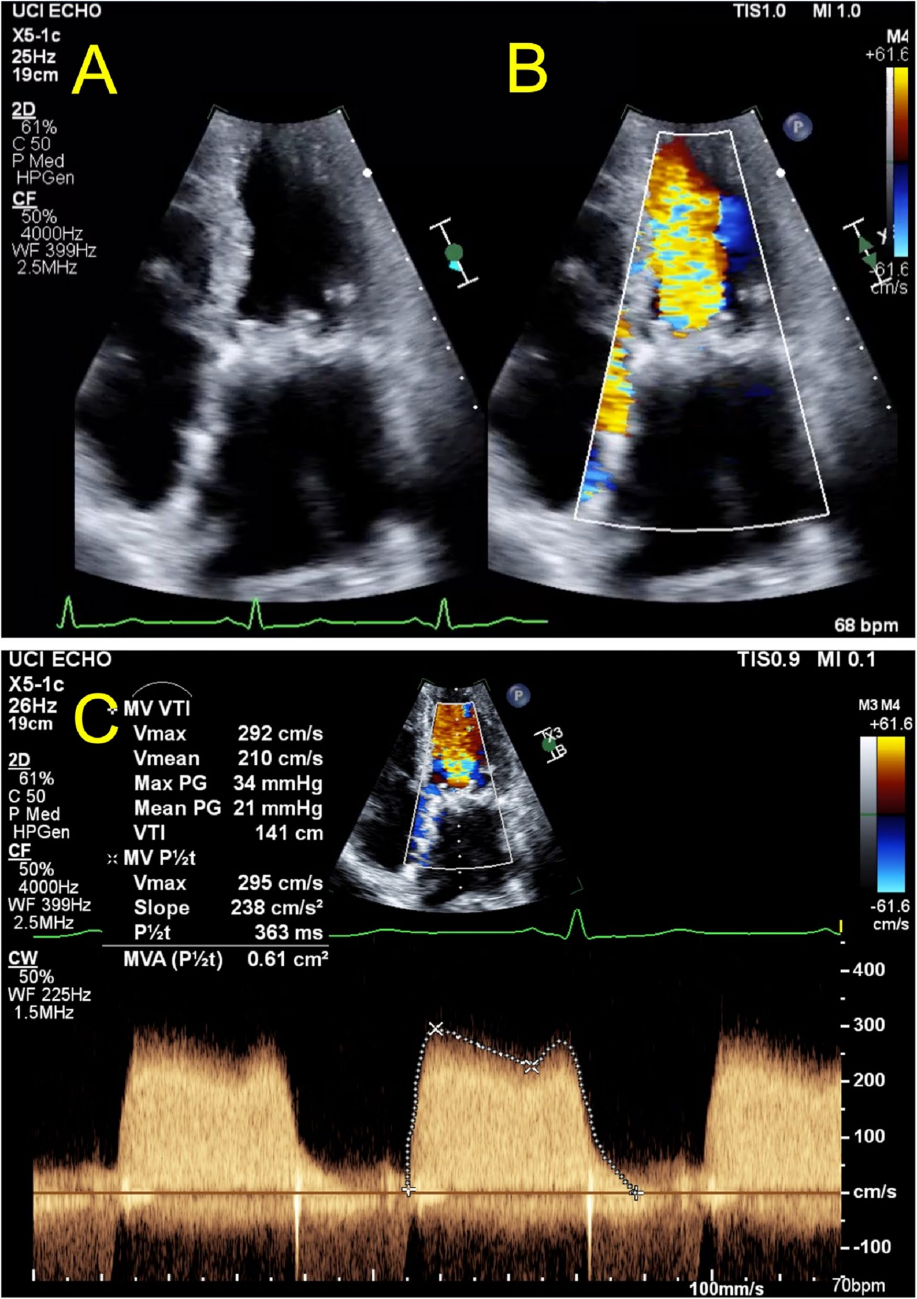
Pre-thrombus echocardiographic images of apical 4 chamber view of bioprosthetic mitral valve. Pre-thrombus transthoracic echocardiogram (TTE) and images of the bioprosthetic mitral (MV) valve in end-diastole demonstrating severely thickened and restricted leaflet motion in the (**A**) apical 4 chamber view without color Doppler, (**B**) apical 4 chamber view with color Doppler showing flow acceleration at the level of the MV annulus and (**C**) continuous wave Doppler tracing with dense envelope with mean gradient of 21 mmHg by velocity-time-integral at a heart rate of 70 beats per minute

**Fig. 4:**
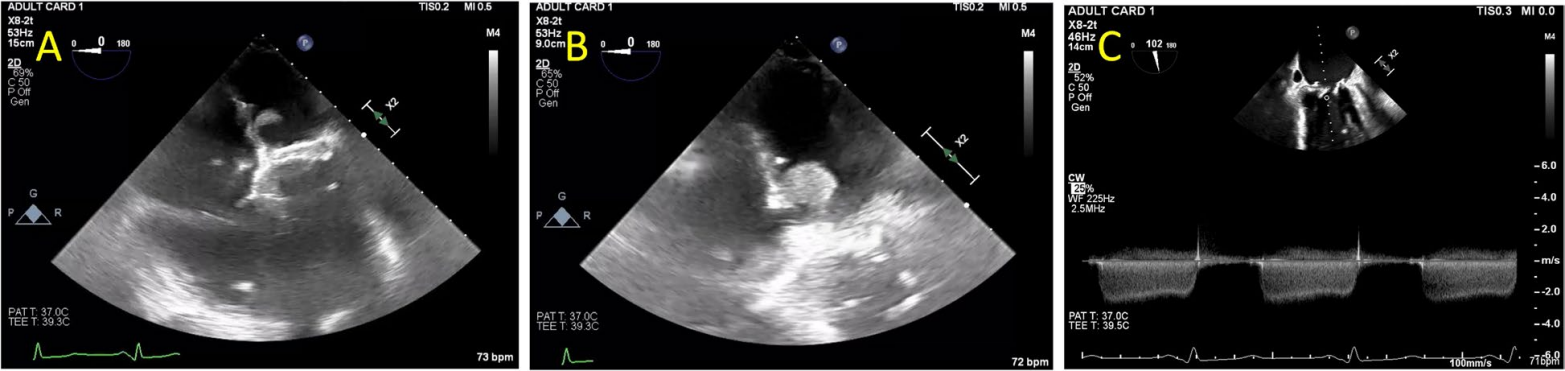
Left atrial thrombus visualized on echocardiogram in mid-esophageal 4 chamber view of bioprosthetic mitral valve. Transesophageal echocardiogram (TEE) demonstrating left atrial thrombus coming into view in end-diastole the (**A**) mid-esophageal 4 chamber view at 0 degrees, (**B**) magnified mid-esophageal 4 chamber view at 0 degrees, and (**C**) continuous wave Doppler across the mitral valve with dense mitral stenosis envelope. Velocity-time integral mean gradient was 19 mmHg at 71 beats per minute

**Fig. 5:**
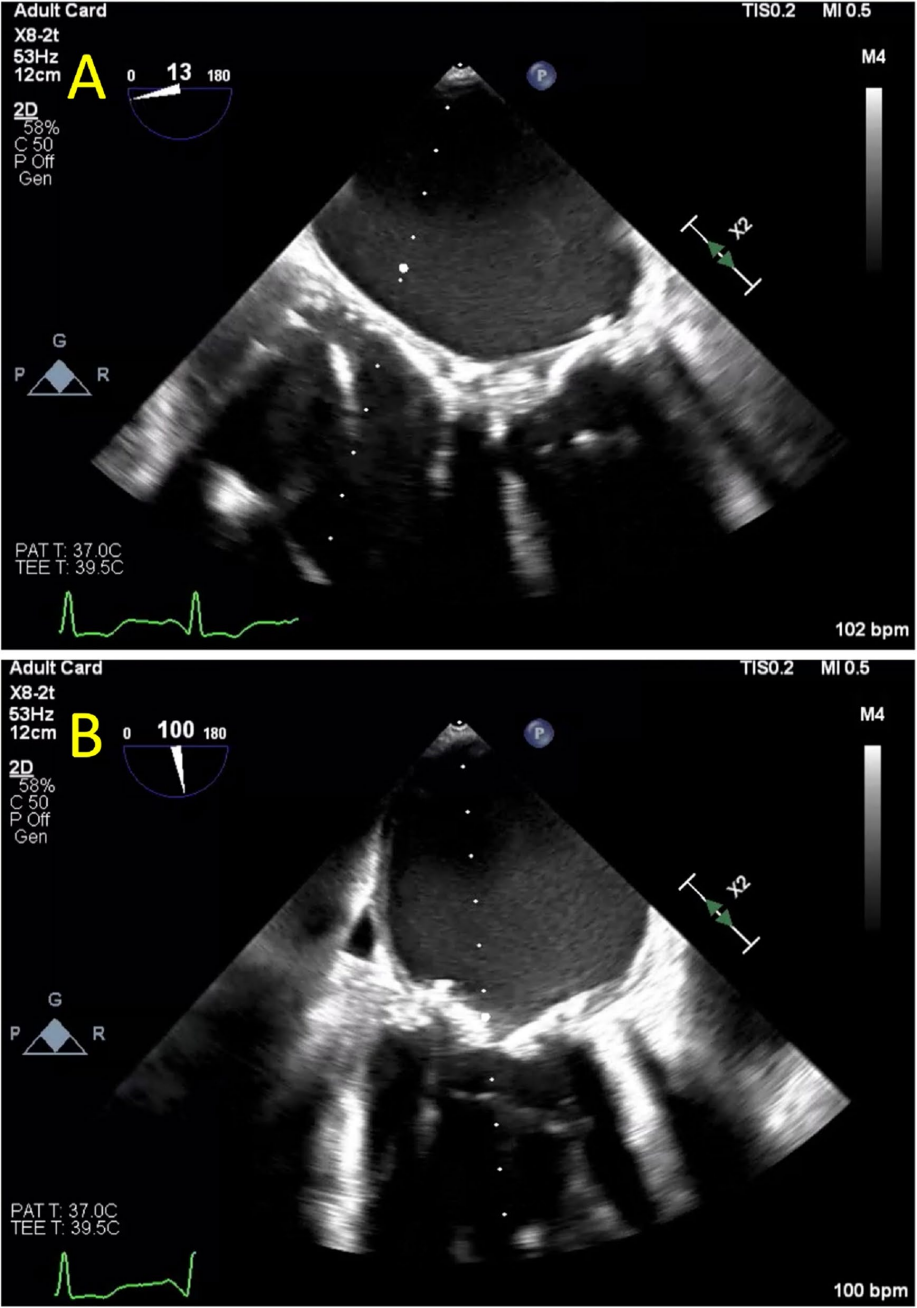
Echocardiographic images of bioprosthetic mitral valve in mid-esophageal 4 chamber and long axis view following left atrial thrombus resolution. Transesophageal echocardiogram (TEE) performed approximately 4 weeks after left atrial thrombus was identified showing resolution of thrombus in end-diastole in the (**A**) mid esophageal 4 chamber view at 13 degrees and (**B**) long axis view at 100 degrees. Severe leaflet restriction is noted, and the mean gradient was measured at 26 mmHg although at a heart rate of 100 beats per minute

At this time an extensive cardiac evaluation including cardiac event monitoring and interim TTE showed an immobile mass on the anterior mitral valve leaflet, which was difficult to discern from a degenerative leaflet. Thus, anticoagulation was initiated empirically to treat valve thrombus. At this time, echocardiographic imaging was not definitively diagnostic of thrombus and leaflet degeneration remained a consideration for which the patient was at risk due to ESRD. The patient continued uninterrupted anticoagulation with warfarin from February 2022 to January 2023 with monitoring of the international normalized ratio (INR) with goal of 2.5 to 3.5 per standard of care. In January of 2023, Transesophageal echocardiography (TEE) revealed progressive worsening of severe bioprosthetic valve stenosis with immobilization of two fused leaflets and mean MV gradient that increased to 21 mmHg (HR 80 bpm, EF noted to be moderately decreased, MV pressure half time 454 msec, MV area 0.5 cm^2^), without evidence of thrombus (Table 1, Figs. 2 and 3). However, the left atrium was noted to be severely dilated with evidence of mild spontaneous echo contrast (SEC). The patient was admitted for definitive inpatient management of the degenerative mitral valve. The MV gradient worsened despite adherence to therapeutic anticoagulation for 11 months, so the etiology for progressive MS was more consistent with leaflet degeneration and fusion of the bioprosthetic MV rather than thrombosis. As the most recent valvular heart disease guidelines do not recommend indefinite anticoagulation for bioprosthetic mitral valve prostheses beyond the first 3 months after implant, warfarin was discontinued in anticipation of procedural intervention [1]. Following these guidelines, heparin bridging was not initiated, as there was no history of atrial fibrillation or other indication for therapeutic anticoagulation [1]. Definitive treatment with transcatheter mitral valve-in-valve replacement was planned after multidisciplinary discussions with the patient and structural heart team. On the morning of the procedure, 8 days after cessation of warfarin, the intra-operative TEE revealed a large 18 mm × 0.9 mm, fixed left atrial thrombus adhered to the left atrial septum, and the procedure was aborted (Fig. 4). The patient made a joint decision with the surgical and cardiology team to defer acute surgical management. He was bridged to warfarin using a heparin infusion with a therapeutic target aPTT of 60–100 seconds with the plan to repeat TEE in 4 weeks and attempt transcatheter mitral valve-in-valve replacement once the left atrial thrombus resolved (Fig. 5).

## Discussion

Our patient had native, non-rheumatic MS likely from multiple comorbidities including ESRD on hemodialysis [2]. Following his bioprosthetic surgical MV replacement during his 4v CABG, he was found to have rapid progression of bioprosthetic MV dysfunction within 2 years of placement with severely elevated trans-mitral gradient and characteristic symptoms of MS. MV replacement either utilizes a bioprosthetic valve, which often uses porcine valves or bovine pericardium, or a mechanical valve which uses a titanium ring with fabric leaflets [3, 4]. Benefits of mechanical valve prosthesis include longer prosthesis durability but requires indefinite anticoagulation with vitamin K antagonists (VKA) [5]. However, the deployment of a mechanical valve would require a surgical approach, which confers a different set of risks to the patient. The decision to proceed with bioprosthetic versus mechanical implants should be made as a joint decision between patient and clinician when possible.

When our patient’s surveillance TTE demonstrated an elevated bioprosthetic mitral valve mean gradient, there was high suspicion for valvular thrombus. The annual incidence of bioprosthetic mitral valve thrombus was previously reported to be close to 0.03% but may be as high as 0.7 to 1.5%. In a 2015 study conducted at Mayo Clinic, 46 of 397 (11.6%, 9 mitral) bioprosthetic valves explanted due to structural failure were found to have deterioration attributed to valvular thrombosis. Of those cases, 65% were detected after the first-year post-procedure, and 15% were found 5 years post-procedure with the majority occurring between 13 to 24 months [6–8]. The rates of valvular deterioration and discovery of valvular thrombus were 16 months and 28 months respectively, similar to prior studies. Typically, bioprosthetic valves last approximately 12–15 years before evidence of deterioration [9]. The recommended frequency of valve surveillance after bioprosthetic mitral valve replacement with a surgical approach is a baseline TTE performed 1–3 months following the intervention, then at 5 and 10 years, and annually after that. However, a transcatheter approach requires TTE to be performed 1–3 months post intervention and then annually [1]. Our patient underwent postoperative TEE. However, due to worsening symptoms within 2 years, he could not reach the five-year surveillance mark. More frequent valvular monitoring in this patient would not have been appropriate prior to the onset of his symptoms.

According to the 2020 American College of Cardiology (ACC)/American Heart Association (AHA) guidelines for management of patients with surgical bioprosthetic valve replacement, monotherapy with aspirin 75 mg to 100 mg daily is adequate after completing 3 to 6 months of anticoagulation without additional indications for continued anticoagulation [1]. For those at low risk of bleeding, the 2020 ACC/AHA guidelines also recommend a vitamin K antagonist (VKA) with an INR goal of 2.5 for at least 3 months and up to 6 months after valve replacement [1]. Similarly, the 2021 European Society of Cardiology (ESC)/European Society of Cardiothoracic Surgery (EACTS) guidelines also state a VKA should be considered in the 3 months following either surgical or transcatheter approaches for patients with mitral valve replacement [10]. For patients with suspected or confirmed valve thrombosis, initial therapy with a VKA is a class 2a recommendation. TTE followed by TEE are class 1 recommendations for the diagnosis of suspected bioprosthetic valve stenosis which were both performed upon symptom onset in our patient. Additionally, the repeat TEE after 9 months of anticoagulation did not show a thrombus of the mitral valve or in the left atrium. Therefore, it was appropriate to discontinue warfarin on admission [1, 10]. There was no other indication for anticoagulation in this patient and a short course of postoperative warfarin therapy had been completed. Once worsening bioprosthetic valvular dysfunction was noted, warfarin was then appropriately resumed empirically for potential thrombus being the source of deterioration although this was not definitive based on imaging. It would be unlikely for a thrombus to persist or for a new clot to form after 11 months of therapeutic anticoagulation with warfarin maintaining therapeutic INR. If mitral valvular atrial fibrillation were present, anticoagulation would have been indicated, but the patient did not have a history of atrial fibrillation, and none was noted on telemetry during his prolonged admission.

It is known that a hypercoagulable state may transiently exist shortly after beginning warfarin which necessitates bridging therapy with heparin. Like novel oral anticoagulants (NOACs), prior studies have also described a potential rebound hypercoagulable period following discontinuing warfarin [11–13]. However, there are no current guidelines for tapering therapy in the period following discontinuation of warfarin. Thrombus formation in our patient may have been due to this post warfarin rebound hypercoagulable period, in the setting of a low-flow state in the left atrium due to mitral stenosis. This etiology is suggested by warfarin being stopped with thrombus discovery only days later; soon after the patient’s INR became subtherapeutic. The rapid progression of valve deterioration was likely multifactorial. Contributing factors likely included his history of end stage renal disease (ESRD) on long term hemodialysis, the presence of a chronic inflammatory state which portends hypercoagulability, and his relatively young age. Prior studies have shown a more rapid deterioration in bioprosthetic valves among patients receiving hemodialysis, but no significant difference has been described between bioprosthetic and mechanical valves [14, 15]. Patients at high risk often have a past medical history satisfying one or all of Virchow’s triad for thrombogenesis which includes groups of endothelial dysfunction, hypercoagulable states, and hemostatic factors [16]. Endothelial damage initiating the platelet adherence/aggregation phase with subsequent activation of the coagulation cascade can occur from trauma, damage to pre-existing plaque, and shearing forces on existing endothelial structures. Hypercoagulable states can include malignancy, genetic hypercoagulability, and inflammatory states such as ESRD, autoimmune conditions (e.g. lupus, anti-phospholipid syndrome), and sepsis. Hemostatic factors can include venous congestion or insufficiency, obstructive or cardiogenic shock, cardiac valvular abnormalities or existing prosthetic valve, cardiac chamber dilation, and arrhythmia including atrial fibrillation. Additionally, an imaging finding known as spontaneous echo contrast (SEC) which appears as “smoke” in the left atrium on TEE is independently associated with a hypercoagulable state and thrombotic risk [17].

The patient’s highest risks for thrombus were existing prosthetic valve with concomitant stenosis, severe left atrial dilation, presence of left atrial SEC, atherosclerosis, ESRD, and peripheral artery disease [18, 19]. Structurally, the patient’s left atrial dilation disturbs blood flow through the chamber, particularly against a stenotic mitral valve, likely contributing to thrombus formation. The finding of left atrial SEC is theorized to be caused by a high inflammatory state leading to increased red blood cell and platelet aggregation indicative of the increased potential for thrombus [17]. However, there wasn’t a direct indication for continued anticoagulation. In the case of mechanical prosthetic valves, patients are bridged after cessation of warfarin prior to operations, as they require indefinite anticoagulation. Patients with non-valvular atrial fibrillation also have indication to use CHA_2_DS_2_VASc score above 5 for bridging to heparin infusion, as elevated scores have been shown to be associated with higher rates of prosthetic valvular thrombus formation [20, 21]. However, our patient was on continuous telemetry without evidence of atrial fibrillation. However, given this patient’s multiple risk factors for clot formation such as ESRD, peripheral artery disease, and severe valvular stenosis, he may have benefited from bridging therapy with a heparin infusion. It is unclear whether bridging after warfarin discontinuation would have prevented thrombus formation but there may be benefit particularly in high-risk patients when transitioning off VKA therapy. It may be prudent to bridge anticoagulation to heparin while waiting for valvular repair, particularly during rebound hypercoagulability following cessation of warfarin.

We encourage additional investigation into the need for further risk stratification to guide anticoagulation recommendations in patients with severe bioprosthetic valve dysfunction with concomitant risk factors for thrombogenesis. Identification of hemodynamic parameters including mean gradients or pressure half-times in MS that increase the risk of prosthetic thrombosis that could be particularly helpful in certain cases is not addressed in current valvular heart disease guidelines. Additionally, the removal of anticoagulation should be carefully considered in patients with such risk factors. Risk stratification could potentially be based on factors such as duration of prior warfarin therapy, severity of trans-mitral gradient, duration of hemodialysis, age of onset of renal failure, and overall risk of thrombus formation accounting for prior history of vasculopathy. Further studies should examine the predictive value of CHA_2_DS_2_VASc for prosthetic valve thrombus in patients with bioprosthetic cardiac valve replacements.

## Conclusions

The etiology of our patient’s progression of bioprosthetic mitral valve disease eventually resulting in thrombus was likely multifactorial in the setting of several comorbidities conferring a prothrombotic state in addition to rebound hypercoagulability following anticoagulation cessation. Patients with dysfunctional mitral valve prostheses may represent a unique population that deserves further consideration for empiric therapeutic anticoagulation, and whose risk of de novo thrombosis may be underappreciated. We suggest nuanced clinical decision-making be applied when considering anticoagulation in patients with bioprosthetic mitral valves who have additional comorbidities present even though they may not meet current guideline criteria. Further investigation into risk stratification of comorbidities may be beneficial to help prevent thrombosis for patients at higher risk.

## Data Availability

All data generated or analyzed during this study are included in this published article.

## Abbreviations

ESRD: End stage renal disease
CAD: Coronary artery disease
CABG: Coronary artery bypass graft
MV: Mitral valve
4v: Four vessel
TEE: Transesophageal echocardiography
EF: Ejection fraction
TTE: Transthoracic echocardiography
NR: International normalized ratio
AHA: American Heart Association
ACC: American college of cardiology
VKA: Vitamin K antagonist
ESC: European society of cardiology
EACTS: European society of cardiothoracic surgery

## References

[1] Otto CM, Nishimura RA, Bonow RO, Carabello BA, Erwin JP, Gentile F (2021). 2020 ACC/AHA guideline for the Management of Patients with Valvular Heart Disease: a report of the American College of Cardiology/American Heart Association joint committee on clinical practice guidelines. Circulation..

[2] Kipourou K, O'Driscoll JM, Sharma R (2022). Valvular heart disease in patients with chronic kidney disease. Eur Cardiol..

[3] van Wachem PB, van Luyn MJA, Buschow KHJ, Cahn RW, Flemings MC, Ilschner B, Kramer EJ, Mahajan S (2001). Collagen Derived Materials. Encyclopedia of materials: science and technology.

[4] Harris C, Croce B, Cao C (2015). Tissue and mechanical heart valves. Ann Cardiothorac Surg..

[5] Hoffmann G, Lutter G, Cremer J (2008). Durability of bioprosthetic cardiac valves. Dtsch Arztebl Int..

[6] Mishra T, Dawdy J, Sood A, Kottam A, Afonso L (2021). Late-onset bioprosthetic mitral valve thrombosis treated with Apixaban. Circ Cardiovasc Imag..

[7] Grunkemeier GL, Li HH, Naftel DC, Starr A, Rahimtoola SH (2000). Long-term performance of heart valve prostheses. Curr Probl Cardiol..

[8] Egbe AC, Pislaru SV, Pellikka PA, Poterucha JT, Schaff HV, Maleszewski JJ (2015). Bioprosthetic valve thrombosis versus structural failure: clinical and echocardiographic predictors. J Am Coll Cardiol..

[9] Applegate PM, Boyd WD, Applegate Ii RL, Liu H (2017). Is it the time to reconsider the choice of valves for cardiac surgery: mechanical or bioprosthetic?. J Biomed Res..

[10] Vahanian A, Beyersdorf F, Praz F, Milojevic M, Baldus S, Bauersachs J (2021). 2021 ESC/EACTS guidelines for the management of valvular heart disease: developed by the task force for the management of valvular heart disease of the European Society of Cardiology (ESC) and the European Association for Cardio-Thoracic Surgery (EACTS). Eur Heart J..

[11] Palareti G, Legnani C, Warfarin withdrawal. (1996). Pharmacokinetic-pharmacodynamic considerations. Clin Pharmacokinet..

[12] Niazi M, Khan D, Mustafa A, Munir AB, Karam B, Snyder ST (2021). Left atrial Thrombus mimicking Myxoma secondary to rebound hypercoagulable state. J Med Cases..

[13] Nagasayi S, Varman S, Ting YY, Ang W (2017). Rivaroxaban withdrawal and rebound hypercoagulability leading to upper extremity deep vein thrombosis: a case report. Age Age..

[14] Kuroda Y, Marui A, Arai Y, Nagasawa A, Tsumaru S, Arakaki R (2021). Impact of dialysis in patients undergoing bioprosthetic aortic valve replacement. Interact Cardiovasc Thorac Surg..

[15] Kim KS, Belley-Côté EP, Gupta S, Pandey A, Alsagheir A, Makhdoum A (2022). Mechanical versus bioprosthetic valves in chronic dialysis: a systematic review and meta-analysis. Can J Surg..

[16] Lowe GD (2003). Virchow's triad revisited: abnormal flow. Pathophys Haemost Thromb..

[17] Ito T, Suwa M (2019). Left atrial spontaneous echo contrast: relationship with clinical and echocardiographic parameters. Echo Res Pract..

[18] Roudaut R, Serri K, Lafitte S (2007). Thrombosis of prosthetic heart valves: diagnosis and therapeutic considerations. Heart..

[19] Gencbay M, Turan F, Degertekin M, Eksi N, Mutlu B, Unalp A (1998). High prevalence of hypercoagulable states in patients with recurrent thrombosis of mechanical heart valves. J Heart Valve Dis..

[20] BRIDGE Study Investigators. Bridging anticoagulation: is it needed when warfarin is interrupted around the time of a surgery or procedure? Circulation. 2012;125(12):e496–8. 10.1161/CIRCULATIONAHA. 10.1161/CIRCULATIONAHA.111.084517 22451610

[21] Çınar T, Hayıroğlu MI, Tanık VO, Aruğaslan E, Keskin M, Uluganyan M (2018). The predictive value of the CHA2DS2-VASc score in patients with mechanical mitral valve thrombosis. J Thromb Thrombol..

